# Telemedicine-based proactive patient management during positive airway pressure therapy: Impact on therapy termination rate

**DOI:** 10.1007/s11818-016-0098-9

**Published:** 2017-01-23

**Authors:** Holger Woehrle, Joachim H. Ficker, Andrea Graml, Ingo Fietze, Peter Young, Helmut Teschler, Michael Arzt

**Affiliations:** 1 Sleep and Ventilation Center Blaubeuren, Respiratory Center Ulm, Olgastr. 83, 89073 Ulm, Germany; 2 ResMed Science Center, ResMed Germany, Martinsried, Germany; 3 Department of Respiratory Medicine, Allergology and Sleep Medicine, General Hospital Nuremberg, Nuremberg, Germany; 4 Paracelsus Medical University, Nuremberg, Germany; 5 Centrum für Herz-Kreislauf- und Gefäßmedizin, Interdisziplinäres Schlafmedizinisches Zentrum, Charité – Universitätsmedizin Berlin, Berlin, Germany; 6 Klinik für Schlafmedizin und Neuromuskuläre Erkrankungen, Universitätsklinikum Münster, Münster, Germany; 7 Department of Pneumology, Ruhrlandklinik, West German Lung Center, University Hospital Essen, University Duisburg-Essen, Essen, Germany; 8 Klinik und Poliklinik für Innere Medizin II, Universitätsklinikum Regensburg, Regensburg, Germany

**Keywords:** Monitoring, ambulatory, Health behavior, Patient adherence, Obstructive sleep apnea, Patient compliance, Ambulantes Monitoring, Gesundheitsverhalten, Patientenadhärenz, Obstruktive Schlafapnoe, Patientencompliance

## Abstract

**Background:**

Adherence to positive airway pressure (PAP) therapy is essential for the benefits of therapy to be realised. Telemedicine-based strategies provide a new option for enhanced monitoring and intervention to promote adherence during PAP. This study investigated the impact of telemedicine-based proactive patient management on PAP therapy termination rates versus standard care.

**Methods:**

Observational data were obtained from ResMed Germany Healthcare, a German homecare provider. Patients were undergoing routine homecare using either a standard or proactive management strategy. The proactive strategy used data from AirView™, a cloud-based remote monitoring system, to prompt patient contact and information sharing/education. Patients receiving their first PAP therapy were included and analysed in matched pairs.

**Results:**

In all, 3401 patients were included in each group. In the first year of PAP therapy, overall therapy termination rate was significantly lower (5.4% vs 11.0%; *p* < 0.001) and time to therapy termination was significantly longer (348 ± 58 vs 337 ± 76 days; *p* < 0.05) in the proactive versus standard care group. Cox proportional hazard analysis revealed a significantly reduced risk of PAP termination in the proactive versus the standard care group (hazard ratio 0.48, 95% confidence interval 0.4–0.57). Findings were consistent in subanalyses according to gender, type of device and insurance status, and in patients aged ≥40 years. However, in the subgroup of patients aged younger than 40 years, the risk of PAP termination was similar in the proactive and standard groups.

**Conclusion:**

A telemedicine-based proactive management strategy compared with standard care of PAP patients was associated with a lower long-term therapy termination rate.

## Zusammenfassung

**Hintergrund:**

Eine ausreichende Therapieadhärenz ist erforderlich, um das therapeutische Potenzial einer Positivdrucktherapie (PAP) auszuschöpfen. Telemedizinbasierte Strategien ermöglichen durch Therapiemonitoring proaktive individuelle und zeitnahe Interventionen, um die PAP-Adhärenz zu steigern. Diese Studie untersuchte den Einfluss der telemedizinbasierten proaktiven Versorgung von Patienten mit Schlafapnoe auf die Abbruchrate der PAP-Therapie.

**Methoden:**

Beobachtungsdaten wurden durch den Deutschen Homecare Provider ResMed Healthcare bereitgestellt. Im Rahmen eines Fall-Kontroll-Designs wurden Patienten, die erstmalig auf eine PAP-Therapie eingestellt wurden, mit PAP-Standardversorgung und proaktiver PAP-Versorgung verglichen. Die proaktive PAP-Versorgung erfolgte mit Hilfe der AirView™-Technologie, einem Cloud-basierten automatisierten Monitoringsystem, das bei Therapieproblemen einen zeitnahen proaktiven Patientenkontakt und Informationsaustausch bzw. eine Patientenschulung einleiten kann.

**Ergebnisse:**

In beide Gruppen wurden jeweils 3401 Patienten eingeschlossen. Innerhalb des ersten Jahres nach der Einstellung auf eine PAP-Therapie war in der Gruppe mit proaktiver PAP-Versorgung im Vergleich zu einer PAP-Standardversorgung die Abbruchrate der PAP-Therapie signifikant niedriger (5,4 % vs. 11,0 %; *p* < 0,001) und die durchschnittliche Zeit bis zum Therapieabbruch signifikant länger (348 ± 58 vs. 337 ± 76 Tage; *p* < 0,05). Diese Verbesserung war unabhängig von Geschlecht, Gerätetyp und Art der Krankenversicherung sowie bei Patienten im Alter von >40 Jahren nachweisbar. In der Gruppe der Patienten unter 40 Jahren war kein Vorteil einer proaktiven Strategie nachweisbar.

**Schlussfolgerung:**

Ein telemedizinbasiertes proaktives PAP-Versorgungsmodell ist mit einer signifikant geringeren Abbruchrate als bei einer konventionellen PAP-Versorgung assoziiert.

## Introduction

Continuous positive airway pressure (CPAP) is the most effective treatment for obstructive sleep apnoea (OSA) and is mandated by current guidelines [1]. However, the benefits of treatment cannot be realised if patients do not adhere to prescribed therapy.

A number of different approaches to improve compliance with CPAP therapy have been assessed in clinical trials, including patient education [2–5], peer support [6, 7], behavioural interventions [3, 4], clinical support [8] and choice of equipment [9–12]. In addition, features of the healthcare system and service/treatment delivery can have an impact on adherence [13]. Despite this, health stakeholders have not traditionally focussed on addressing adherence issues, and there are relatively few data on interventions to improve adherence, which in turn could improve patient outcomes [13].

Regular follow-up by clinicians and objective assessment of device data have been suggested to be essential in facilitating good compliance with CPAP therapy for OSA [14]. However, current management of CPAP compliance is reactive. Patients who are experiencing challenges or difficulties during CPAP therapy have to contact their healthcare provider to request assistance. Alternatively, less motivated patients might just stop using therapy altogether if there is a problem, or if they perceive there to be a problem.

Telemedicine is a term used to describe the use of information and communications technology to deliver healthcare at a distance [15]. It also offers the opportunity to provide quality care without dramatically escalating healthcare costs [16]. It is especially suited to the management of chronic diseases that require long-term monitoring to ensure successful therapy [17]. Having remote access to CPAP therapy data creates an opportunity for healthcare professionals to intervene proactively and provide appropriate support to individual patients with the aim of having them continue to use PAP therapy. Remote data are useful for tracking CPAP adherence and can also provide information on mask leak and residual apnoea–hypopnoea index (AHI), but there are few data on how use of these systems impacts on outcomes in OSA patients [18].

This study used data from a large German homecare provider to investigate the impact of a proactive patient management programme supported by remote access to PAP therapy data on therapy termination compared with standard care.

## Methods

Study data were obtained from ResMed Germany Healthcare, a German homecare provider. German data protection law allows for the use of such data, if anonymised, for scientific purposes. Therefore, patient informed consent was not necessary in this study.

### Subjects

Patients who started PAP therapy for the first time between 1 September 2009 and 30 April 2014 and were being treated with CPAP, automatic continuous positive airway pressure (APAP), bilevel PAP or adaptive servo-ventilation (ASV) devices, administered via a nasal mask, nasal pillows or full face mask interface, were eligible for inclusion in this analysis.

Patients who had started PAP therapy ≥1 year previously were included. Those who were managed using a proactive, telemedicine-based strategy were matched with PAP users managed using standard care. Patients were excluded from the analysis if they had terminated PAP therapy for a reason that was not related to PAP compliance (e. g. their insurer refused to cover PAP therapy, patient death, transfer to ventilation device). The proactive and standard care cohorts were matched for gender, insurance (public versus private), age (10-year bands), first device used (CPAP, APAP, bilevel PAP or ASV), therapy start date, initial interface (nasal mask, nasal pillows or full face mask) and introduction to device and interface (in sleep laboratory, at home by the homecare provider or at the homecare provider’s service centre).

### Patient management

Standard care for each patient varied depending on their health insurance, but generally included polygraphy after about 6 months and when clinically necessary thereafter, and or patient contact with questions about device usage. If issues arose during PAP therapy the patient was advised to contact his/her healthcare provider, sleep laboratory and/or treating physician. Depending on their insurer, standard care patients might be contacted at 6 months to provide device usage data.

The proactive management strategy was facilitated by data obtained via AirView™, a cloud-based remote monitoring system that connects wirelessly with the PAP therapy devices used by ResMed Germany Healthcare. If AirView™ data showed that compliance during the first 2 weeks of PAP therapy did not meet the required level (average <4 h/day), patients received a telephone call from the homecare provider. For the period from 2 weeks to 6 months, average usage was checked every 14 days and patients were called again each time continued periods of no or low (<4 h/day) usage were identified from AirView™ data. From 6 months of therapy onwards, patients were notified via telephone call or letter if AirView™ data showed that PAP device usage dropped significantly or did not reach the required threshold (based on the insurance-specified usage criteria for reimbursement [approximate average of 4 h/night]). When contacted, patients were proactively provided with detailed information on use of their PAP therapy device and management of side effects (e. g. upper airway dryness, pressure, ulcers).

### Assessments

Patient characteristics, therapy termination rates and time to therapy termination were calculated for the standard care and proactive strategy groups based on data extracted from patient records held by ResMed Germany Healthcare, a German homecare provider. Therapy termination was then described as compliance related if it occurred as the result of patient decision or behavior, was related to complaints by the patient, or was related to the PAP interface (i. e. not accepting or tolerating PAP therapy). Terminations that were not compliance related occurred when a patient was lost to follow-up, transferred to a ventilation device or died, or were related to insurance coverage issues or patient transfer to another homecare provider.

### Data analysis

Descriptive statistics are shown as absolute and relative frequency or mean ± standard deviation (SD), as appropriate. Numerical differences between groups were analysed using t‑tests because the sample size allows for this approximation. For categorical data, differences in proportions were analysed using Z‑tests.

Matching was used to eliminate the influence of unequal distributions of risk factors on differences in compliance between the proactive care and standard care groups. Propensity score matching was conducted based on the following factors: gender, insurance, age (10-year bands), first device, therapy start date, first mask and therapy set up. Time to therapy termination was analyzed using Cox proportional hazards regression with standard vs. proactive care included as an explanatory factor. The results for marginal times to termination were visualized using Kaplan–Meier plots. A *p*-value of <0.05 was considered statistically significant. All statistical analyses were performed using IBM SPSS Statistics 22 and R version 3.0.2.

## Results

A total of 3401 patients who were managed using the proactive strategy and 3401 matched patients receiving standard care were included in the analysis. There were no significant differences between the matched groups at baseline (Table 1).

**Table 1:** Patient demographic and clinical characteristics at baseline

	Standard management (*n* = 3401)	Proactive strategy (*n* = 3401)	*p*-value
Male, *n* (%)	2543 (75)	2527 (74)	0.680
Age, years	59 ± 13	59 ± 13	0.254
*Age group, n* (%)			
<30 years	46 (1)	44 (1)	0.920
30–40 years	210 (6)	215 (6)	0.840
40–50 years	601 (18)	608 (18)	0.850
50–60 years	963 (28)	962 (28)	1.000
60–70 years	816 (24)	800 (24)	0.670
70–80 years	670 (20)	676 (20)	0.880
>80 years	95 (3)	96 (3)	1.000
*First mask, n* (%)			
Nasal	1653 (49)	1659 (49)	0.900
Nasal pillows	462 (14)	451 (13)	0.720
Full face	1286 (38)	1291 (38)	0.920
*First device, n* (%)			
CPAP	1283 (38)	1272 (37)	0.800
APAP	1834 (54)	1855 (55)	0.630
ASV	158 (5)	157 (5)	1.000
Bilevel	126 (4)	117 (3)	0.600
*Insurance, n* (%)			
Public	2902 (85)	2904 (85)	0.970
Private	499 (15)	497 (15)	0.970

*Pairwise testing was performed, level of significance *p* < 0.05

Values are mean ± standard deviation, or number of patients (%)

*APAP* automatic continuous positive airway pressure, *ASV* adaptive servo-ventilation, *Bilevel* bilevel positive airway pressure, *CPAP* continuous positive airway pressure, *PAP* positive airway pressure

### PAP termination

**Fig. 1:**
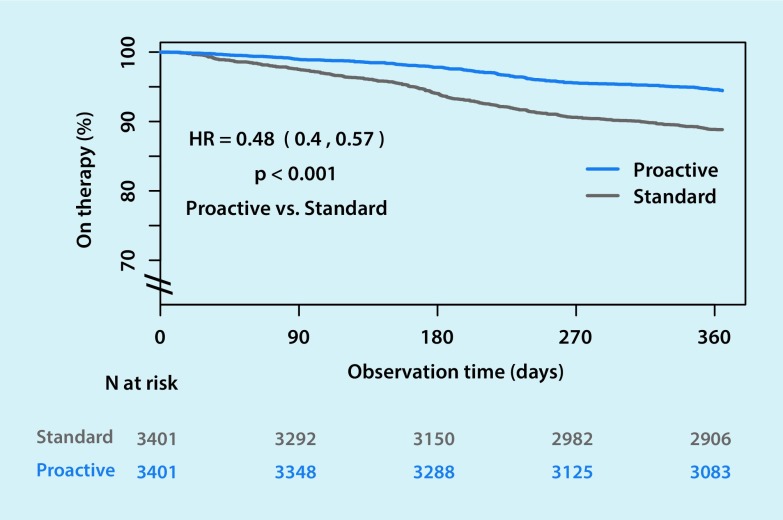
Kaplan–Meier plot showing risk of therapy termination in positive airway pressure users managed using a proactive strategy versus standard care

The overall therapy termination rate was more than halved by the proactive strategy compared with standard care (5.4% vs 11.0%, respectively) (Table 2). Mean time to therapy termination in the first year was 348 ± 58 days in the proactive strategy group compared with 337 ± 76 days in the standard care group. Cox proportional hazard analysis revealed a significantly reduced risk of PAP termination in the proactive versus the standard care group (hazard ratio 0.48, 95% confidence interval 0.4–0.54) (Fig. 1).

**Table 2:** Therapy termination rates at 1 year

	Risk of therapy termination, *n* (%)		Reduction in risk of therapy termination for proactive vs. standard management (%)	*p*-value
	Standard management	Proactive strategy		
Overall	373/3401 (11.0)	185/3401 (5.4)	50.4	<0.001
Male	262/2543 (10.3)	130/2527 (5.1)	50.1	<0.001
Female	111/858 (12.9)	55/874 (6.3)	51.4	<0.001
APAP/CPAP	350/3117 (11.2)	177/3127 (5.7)	49.6	<0.001
ASV/Bilevel	23/284 (8.1)	8/274 (2.9)	63.9	0.011
Public	335/2902 (11.5)	163/2904 (5.6)	51.3	<0.001
Private	38/499 (7.6)	22/497 (4.4)	42.1	0.034

*APAP* automatic continuous positive airway pressure, *ASV* adaptive servo-ventilation, *Bilevel* bilevel positive airway pressure, *CPAP* continuous positive airway pressure

### Subanalyses according to potential modifiers

**Fig. 2:**
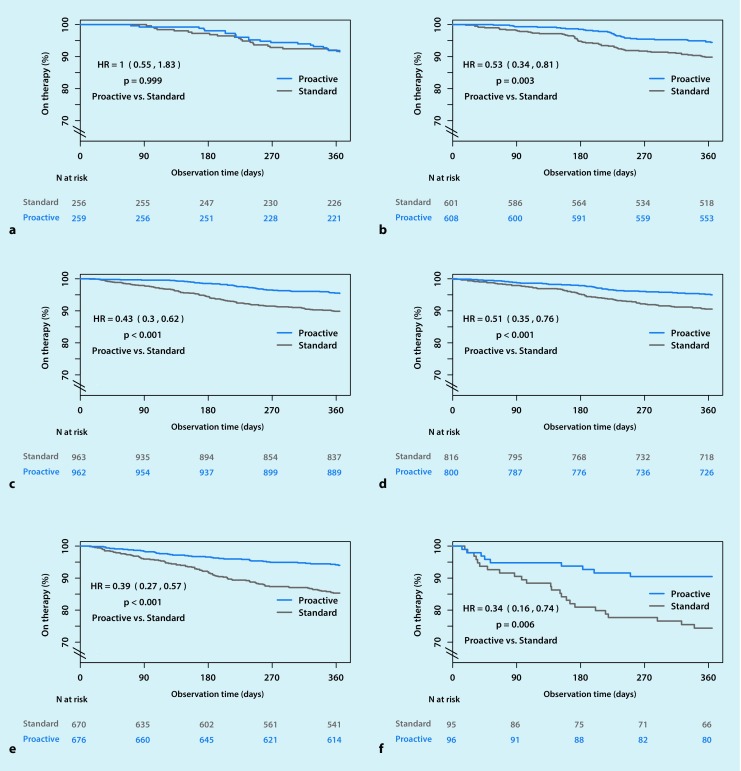
Kaplan–Meier plot showing risk of therapy termination in positive airway pressure users managed using a proactive strategy versus standard care based on patient age (**a** Age <40 years. **b** Age 40–49 years. **c** Age 50–59 years. **d** Age 60–69 years. **e** Age 70–79 years. **f** Age ≥80 years)

Therapy termination rates with the standard and proactive strategies were similar in males and females (Table 2), and for both genders the proactive strategy significantly reduced the risk of therapy termination compared with standard management. The risk of therapy termination was significantly reduced by the proactive strategy versus standard care in patients aged ≥40–69 years and ≥70 years, but there was no significant difference between the two management strategies in younger patients (age <40 years) (Fig. 2). Therapy termination risk reduction with proactive management was the greatest (63.9%) in patients receiving bilevel PAP or ASV, although the risk of therapy termination did not differ significantly based on the type of device used (Table 2). The overall significant benefit of the proactive strategy compared with standard care on therapy termination was the same in patients whose first device was APAP or CPAP, or bilevel or ASV. The proactive strategy was also effective in reducing the therapy termination rate in patients with either public or private insurance, with a slightly greater beneficial effect in those with public insurance.

## Discussion

The results of this study showed that a telemedicine-based proactive strategy for the management of patients receiving PAP therapy reduced the rate of therapy termination over time, irrespective of gender, device used and the type of insurance. The finding that the proactive strategy was effective irrespective of the type of PAP device used might suggest that therapy acceptability (e. g. interface comfort) may play a bigger role than the type of device being used. Interestingly, the positive effects of proactive care were seen in all age groups except those aged <40 years. One possible explanation for this may be that the youngest patients are more comfortable with using information technology to better inform themselves about their condition.

To the best of our knowledge, this study is the first to investigate the impact of a telemedicine-based strategy on long-term continuation of PAP therapy. The main outcome measure in many previous studies has been compliance in terms of hours of device usage per night, and there is a lack of data on therapy termination rates. The majority of these studies have focussed only on CPAP in patients with OSA, used a variety of different telemedicine-based strategies, and reported inconsistent results. In addition, the majority were small, single-centre and/or short-term studies [19–24].

Interface problems and side effects are the most common causes of CPAP intolerance and an important potential benefit of telemedicine-based strategies is early identification of these issues and the implementation of strategies to resolve them in a timely fashion. Other benefits of telemonitoring strategies have been reported, such as reduced labour time for healthcare staff, allowing the same levels of adherence and efficacy to be achieved with fewer staff resources [17, 25]. This is important in the context of a projected increase in the number of patients presenting with sleep-disordered breathing due to the aging population and the growing rates of comorbidities such as obesity and diabetes that will increase demand for already stretched healthcare services. The ability to utilise telemedicine to provide patient-focussed, proactive and intensive management of patients using PAP therapy facilitates the management of large patient populations, as well as significantly improving therapy continuation rates and usage. Telemedicine also appears to be a cost effective strategy for managing CPAP treatment in patients with OSA [16].

Improved patient engagement is another potential advantage of telemedicine strategies, which in turn could contribute to additional gains in terms of PAP adherence and outcomes. The positive impact of patient involvement in therapy was highlighted in a recent study which showed that providing patients with web-based access to their personal PAP usage data increased device usage in the first week of therapy and over 3 months of follow-up compared with usual care [26].

According to the World Health Organisation (WHO), it is important that health systems evolve to meet new challenges [13]. The introduction of new technologies, such as telemedicine, is one way in which healthcare systems and providers can advance patient care. In the case of PAP therapy this could contribute to better adherence and improved patient outcomes. WHO has also recognised that interventions targeted at improving adherence should provide a significant positive return on investment via prevention of adverse health outcomes, and that there are also economic benefits associated with improved quality of life, including reductions in indirect costs and increased productivity [13]. In the context of CPAP therapy for OSA, the importance of increasing adherence has also been recognised by a Cochrane review that emphasised the need for new strategies to promote CPAP compliance [27].

One of the main limitations of this analysis is the lack of specific data on device usage in the standard care group. Thus, we report therapy termination rates rather than device usage hours, which made comparison with existing data more difficult. However, it could be argued that therapy termination is the most important endpoint to assess because once a patient stops therapy getting them to restart is likely to be difficult. In contrast, reduced usage provides a cue for intervention, and there is always the possibility that hours of device use will increase. Another potential limitation is that the ability to accurately determine therapy termination is different in the two groups – it is very obvious when patients in the proactive strategy group stop PAP therapy based on device data received, but therapy termination might be less obvious in the standard care group for whom the requirement for a homecare provider visit may be the only indicator of therapy problems or termination. Interrogation of big data allows inclusion of a very large population of patients treated in routine clinical practice. However, databases created for administrative, rather than scientific, purposes do not include a full set of clinical data. In the current analysis, this means that we had no data on clinical features, insomnia and other factors that might have influenced the outcomes under study. Furthermore, these are observational data and therefore do not provide proof for a causative relationship between the intervention (proactive care) and the outcome (reduced therapy termination). Data from randomised clinical trials are needed to more clearly elucidate the effects of a telemedicine-based proactive management strategy on PAP therapy termination rates. In addition, future research should further investigate the potential of telemonitoring-guided proactive care on additional endpoints, including device usage, resource use and healthcare professional time requirements.

## Conclusion

This analysis of a large population of PAP therapy users in Germany showed that the long-term rate of therapy termination was significantly lower in patients managed using a telemedicine-based proactive management strategy compared with standard care. This association was observed in both men and women, across a wide range of patient ages, for all PAP devices and in patients with public or private insurance. Implementation of telemedicine-based strategies has the potential to improve adherence and patient outcomes and may allow more efficient allocation of scarce healthcare resources. Randomized, controlled clinical trials are needed to further assess both therapy termination rates and device usage, which would complement our large data set from routine clinical practice.
